# Emphysema phenotypes and lung cancer risk

**DOI:** 10.1371/journal.pone.0219187

**Published:** 2019-07-25

**Authors:** Jessica González, Claudia I. Henschke, David F. Yankelevitz, Luis M. Seijo, Anthony P. Reeves, Rowena Yip, Yiting Xie, Michael Chung, Pablo Sánchez-Salcedo, Ana B. Alcaide, Aranzazu Campo, Juan Bertó, María del Mar Ocón, Jesus Pueyo, Gorka Bastarrika, Juan P. de-Torres, Javier J. Zulueta

**Affiliations:** 1 Pulmonary Service, Clínica Universidad de Navarra, Pamplona, Spain; 2 Department of Radiology Mount Sinai School of Medicine, NY, United States of America; 3 School of Electrical and Computer Engineering, Cornell University, Ithaca, NY, United States of America; 4 D4Vision, Inc, Ithaca, NY, United States of America; 5 Pulmonary Service, Complejo Hospitalario de Navarra, Pamplona, Spain; 6 Radiology Department, Clínica Universidad de Navarra, Pamplona, Spain; 7 Navarra’s Health Research Institute (IDISNA), Pamplona, Spain; 8 CIBERONC, ISCIII, Madrid, Spain; 9 VisionGate, Inc, Phoenix, Arizona, United States of America; University of Pittsburgh, UNITED STATES

## Abstract

**Background:**

To assess the relationship between lung cancer and emphysema subtypes.

**Objective:**

Airflow obstruction and emphysema predispose to lung cancer. Little is known, however, about the lung cancer risk associated with different emphysema phenotypes. We assessed the risk of lung cancer based on the presence, type and severity of emphysema, using visual assessment.

**Methods:**

Seventy-two consecutive lung cancer cases were selected from a prospective cohort of 3,477 participants enrolled in the Clínica Universidad de Navarra’s lung cancer screening program. Each case was matched to three control subjects using age, sex, smoking history and body mass index as key variables. Visual assessment of emphysema and spirometry were performed. Logistic regression and interaction model analysis were used in order to investigate associations between lung cancer and emphysema subtypes.

**Results:**

Airflow obstruction and visual emphysema were significantly associated with lung cancer (OR = 2.8, 95%CI: 1.6 to 5.2; OR = 5.9, 95%CI: 2.9 to 12.2; respectively). Emphysema severity and centrilobular subtype were associated with greater risk when adjusted for confounders (OR = 12.6, 95%CI: 1.6 to 99.9; OR = 34.3, 95%CI: 25.5 to 99.3, respectively). The risk of lung cancer decreases with the added presence of paraseptal emphysema (OR = 4.0, 95%CI: 3.6 to 34.9), losing this increased risk of lung cancer when it occurs alone (OR = 0.7, 95%CI: 0.5 to 2.6).

**Conclusions:**

Visual scoring of emphysema predicts lung cancer risk. The centrilobular phenotype is associated with the greatest risk.

## Introduction

Studies exploring the association between lung cancer and chronic obstructive pulmonary disease (COPD) have focused on airway obstruction (AO) or on self-reported chronic bronchitis or emphysema [1–4]. A recent analysis of the National Lung Screening Trial (ACRIN), reported a strong linear relationship between increasing severity of airflow obstruction and lung cancer risk, while several lung cancer screening cohorts have reported an association between emphysema and lung cancer.[5,6] In the latter, AO and the presence of emphysema on LDCT were associated with a two-to-threefold increase in lung cancer risk. After adjusting for confounders such as tobacco exposure, age, sex, and the presence of AO, emphysema is the strongest predictor of risk, even in non-smokers.[7–9] When comparing the impact of emphysema in the context of lung cancer screening in current, former and never smokers exposed to second hand smoke, the risk of lung cancer in never smokers with emphysema is similar to that in smokers (2.1% and 2.6%, respectively, *p* = 0.61), and 6-fold higher than in never smokers without emphysema.[10].

The association between emphysema and lung cancer risk has been significant primarily in studies in which visual assessment, as opposed to computer assessment, was performed.[5,6, 11–15] No study, however, has investigated the contribution of different emphysema phenotypes to lung cancer risk. Centrilobular emphysema (CLE) is associated with smoking and its distribution mimics that of lung cancer including upper lobe predominance.[16] We studied the relationship between lung cancer risk, AO and emphysema phenotype and severity using widely accepted visual scoring methods.

## Materials and methods

### Participants

Study subjects were enrolled in our lung cancer screening program between September 2000 and December 2016.[17] Inclusion criteria were age > 40 years, and a cumulative tobacco exposure of ≥ 10 pack-years. Patients with lung cancer symptoms, or a history of other cancers within 5 years were excluded from screening. The screening protocol was approved by the Research Ethics committee of Navarra University (project number 028/2012 mod 1, entitled “I-ELCAP”) and all subjects signed an informed consent prior to enrollment. Details of the screening protocol may be found in www.ielcap.org.

A total of 73 patients were diagnosed with lung cancer among 3,477 subjects enrolled in the screening program. One patient was excluded from the present study because imaging studies were not technically analyzable. Lung cancer was diagnosed on baseline or annual screenings in 30 and 42 participants respectively, with a mean of 40.7 months between the first visit and the cancer diagnosis. After adjusting for sex, age, pack-years, and smoking status, 216 individuals from the screened cohort were selected as controls with a 1:3 ratio of lung cancer cases to controls.

### Pulmonary function tests

Pulmonary function tests (PFT) were performed with a flow spirometer (Vmax22; SensorMedics, Yorba Linda, CA) according to ATS guidelines.[18] Post-bronchodilator values were expressed as a percentage of the predicted value according to the European Community Lung Health Survey.[19] The presence and severity of AO was determined using criteria of the Global Initiative for Chronic Obstructive Lung Disease (GOLD; forced expiratory volume in 1 s/forced vital capacity (FEV1/FVC) ratio <70%) [20].

### Low dose chest CT (LDCT)

Eleven cases and 19 controls included in this study underwent single-slice helical scanning (Somatom Plus 4; Siemens; Erlangen, Germany) at low-dose settings (140 kVp, 43 mAs) and 1.5 pitch with a collimation of 8 mm, providing. The rest of the cohort was scanned using a sixty-four slice multidetector CT scanner (Somatom Sensation 64, Somatom Definition, Siemens Healthcare, Erlangen, Germany) at a low-dose setting (120 kV tube voltage, 40 mAs tube current, 64x0.6 mm slice collimation, 0.5 s gantry rotation time, 1.4 pitch, 1 mm slice thickness, 1 mm reconstruction interval). Examinations were acquired with patients in the supine position, in cranio-caudal direction and at end-inspiration. Resulting images were reconstructed with a high convolution reconstruction algorithm (B60) and lung window [21].

### Assessment of emphysema on LDCT

A pulmonologist (JG) visually scored the baseline LDCT for emphysema presence, type and severity, using validated criteria established by the Fleischner Society.[22] In general, pulmonary emphysema was classified into the following subtypes: centrilobular (CLE), panlobular (PLE) and paraseptal (PSE). There were no individuals with bullous or advanced destructive emphysema in our cohort. Only one subject was classified as PLE and was excluded from the analysis. For the purpose of data analysis, subjects were assigned to three categories: 1) No emphysema, 2) CLE and 3) PSE. Scoring procedures used a five-level semiquantitative scale based on criteria used in the National Emphysema Treatment Trial.[23] However, no division of the lung into different zones was undertaken. Severity was assessed using this scoring system throughout the whole lung.

For internal quality control, all 72 cases and 50 random controls were also independently reviewed by a radiologist (MC). Kappa statistics for the reading agreement between the pulmonologist (JG) and the radiologist was 0.87 (*p*<0.00001). Both readers (JG and MC) were blinded to lung cancer diagnosis when evaluating emphysema presence and severity.

### Statistics

Statistical analysis was performed using the matched set of seventy-two cancer cases and 216 control subjects. Conditional logistic regression (SAS version 9.4; SAS Institute, Cary, NC) was used to assess whether %predicted FEV1 or FEV1/FVC, were risk factors for lung cancer. Lung function related variables were studied as continuous and ordinal variables, using the GOLD classification for FEV1: I: ≥80%; II: 50–80%; II: 30–50% and IV: <30%. Twelve patients were reclassified into a different GOLD group when using the post-bronchodilator FEV1. Additional analyses were performed adjusting for GOLD classification, visual emphysema and smoking status.

## Results

Baseline characteristics of the 72 study patients with lung cancer and the 216 screened controls without lung cancer are shown in Table 1. As expected, there were no significant differences between subjects in average age and average pack-years of smoking. There were, however, more current smokers in the group of cases with cancer (73.6% vs 52.1%, *p* = 0.0014) and for this reason all analyses were adjusted by smoking status.

**Table 1:** Patient demographics.

			N = 287
Demographic	Case Subjects (n = 72)	Control Subjects (n = 215)	p-value
Sex, No. (%)			0.99
Female	12 (16.7)	36 (16.7)	
Male	60 (83.3)	179 (83.3)	
Age, y			0.83
Mean (SD)	63.8 (9)	63.6 (8.8)	
Pack-years of smoking			0.64
Mean (SD)	53.0 (25)	51.5 (22.9)	
Smoking status, No. (%)			0.0014
Active smokers	53 (73.6%)	112 (52.1%)	
BMI			0.46
Mean (SD)	26.7 (4.7)	27.2 (3.9)	
Airflow obstruction, No. (%)			
None	33 (45.8)	150 (69.8)	0.0004
GOLD I	27 (37.5)	36 (16.3)	
GOLD II	9 (12.5)	27 (12.6)	
GOLD III-IV	3 (4.2)	3 (1.4)	
Emphysema, No. (%)	59 (81.9)	90 (41.8)	<0.001
CLE, No. (%)	30 (50.8)	9 (10)	<0.001
PSE, No. (%)	2 (3.4)	31 (34.4)	
CLE and PSE, No. (%)	27 (45.8)	50 (55.5)	

*Definition of abbreviations*: SD = Standard deviation; BMI = body mass index; GOLD = Global Initiative for Chronic Obstructive Lung Disease; CLE = Centrilobular; PSE = Paraseptal.

Adenocarcinoma (50%) was the most frequent type of lung cancer, followed by squamous cell carcinoma (20.8%). Other cell types included small cell carcinoma (6.9%), large cell carcinoma (9.7%), neuroendocrine tumors (5.6%), and mixed tumors (4.2%). Histology was unknown in two patients. Six patients had a second primary lung cancer identified on subsequent screening rounds. Staging based on clinical assessment or pathologic findings when available, was distributed as follows: 71% (51/72) in stage I, 8.3% (6/72) in stage II, 9.72% (7/72) in stage III and 11.11% (8/72) in stage IV.

Conditional logistic regression was used to determine whether airflow obstruction and the presence of radiographic emphysema are predictors of lung cancer risk independent of age, sex and smoking history (Table 2). Moreover, we performed an interaction model to try to clarify the relationship of the different emphysema subtypes and lung cancer (Table 3). Due to differences between the groups, all analyses were adjusted by smoking status.

**Table 2:** Multivariate conditional logistic results.

			Stratified on matching pairs*		Adjusted for emphysema*		Adjusted for airflow obstruction*	
	Case Subjects (n = 72)	Control Subjects (n = 215)	OR	95% CI	OR	95% CI	OR	95% CI
**Pulmonary function test**								
**Airflow obstruction**			**(0.0006)**		(0.1244)			
None	33	150	Ref.		Ref.			
Yes	39	65	**2.85**	**1.56–5.21**	1.70	0.86–3.34		
			**(0.0020)**		(0.1026)			
None	33	150	Ref.		Ref.			
GOLD I	27	35	3.77	1.85–7.68	2.50	1.13–5.51		
GOLD II	9	27	1.65	0.68–3.96	0.89	0.34–2.33		
GOLD III-IV	3	3	4.13	0.65–26.3	1.56	0.20–11.86		
**Visual emphysema**								
**Emphysema**			**(<0.0001)**				**(<0.0001)**	
None	13	125	Ref.				Ref.	
Any	59	90	**5.94**	**2.90–12.17**			**5.44**	**2.61–11.35**
**NETT classification**			**(<0.0001)**				**(<0.0001)**	
0	13	125	Ref.				Ref.	
1	47	78	5.39	2.59–11.24			5.16	2.44–10.91
2	9	10	9.64	3.15–29.51			8.72	2.72–27.97
3	3	2	**15.32**	**2.14–109.71**			**12.56**	**1.58–99.90**

Definition of abbreviations: CI = confidence interval; SD = standard deviation; GOLD = Global Initiative for Chronic Obstructive Lung Disease; OR = odds ratio; NETT = National Emphysema Treatment Trial

* All adjusted by smoking status

**Table 3:** Multivariate conditional logistic results, emphysema types.

			Adjusted for airflow obstruction*	
	Case Subjects (n = 72)	Control Subjects (n = 216)	OR	95% CI
**Type of emphysema**			**(<0.0001)**	
None	13	125	Ref.	
CLE	30	9	**34.27**	**25.48–99.31**
CLE and PSE	27	50	**4.02**	**3.61–34.97**
PSE	2	31	**0.68**	**0.54–2.64**
			**(0.094)**	
PSE by CLE Interaction	27	21	**0.17**	**0.03–1.35**

Definition of abbreviations: CI = confidence interval; SD = standard deviation; GOLD = Global Initiative for Chronic Obstructive Lung Disease; OR = odds ratio; NETT = National Emphysema Treatment Trial; CLE = Centrilobular; PSE = Paraseptal

* All adjusted by smoking status

When FEV1 was analyzed as a continuous variable, the postbronchodilator percent predicted FEV1 (PFEV1%) was significantly associated with lung cancer risk (OR = 1.2, 95% CI: 1.0–1.4, *p* = 0.02). When spirometric severity by GOLD classification was used, only GOLD I remained significantly associated with lung cancer risk (Table 4).

**Table 4:** Univariate conditional logistic regression results.

Pulmonary function test					
Measure	Case Subjects (n = 72)	Control Subjects (n = 215)	OR	95% CI	p-value
**PFEV1, % predicted**					**0.02**
Mean (SD)	87.9 (19)	94.2 (19.5)	1.2	(1.0–1.4)	
81+	51 (70.8%)	161 (74.9%)	Ref.		
51–80	17 (23.6%)	38 (17.7%)	1.5	(0.7–2.9)	
31–50	3 (4.2%)	5 (2.3%)	2.1	(0.4–11)	
≤ 30	1 (1.4%)	11 (5.1%)	0.3	(0.04–2.3)	
**PFEV1/FVC**					**0.01**
Mean (SD)	66.6 (10.3)	70.6 (10.3)	1.6	(1.2–2.0)	
71+	30 (41.7%)	136 (63.3%)	Ref.		
61–70	24 (33.3%)	40 (18.6%)	3.2	(1.6–6.5)	
51–60	10 (13.9%)	13 (6%)	3.9	(1.5–10.2)	
≤ 50	8 (11.1%)	26 (12.1%)	1.6	(0.6–4.0)	

Definition of abbreviations: CI = confidence interval; OR = odds ratio; FEV1 = forced expiratory volume in 1 s; FVC = forced vital capacity; P = postbronchodilator

The presence of visually determined emphysema on the baseline chest CT was significantly associated with an increased risk of lung cancer (OR = 5.9, 95% CI: 2.9–12.7; *p*<0.0001). Moreover, this risk was related to the severity of visual emphysema quantified according to the NETT classification: for NETT values of 1 to 3, the OR was 5.4 (95% CI: 2.6–11.2), 9.6 (95% CI: 3.2–29.5) and 15.3 (95% CI: 2.1–109.7), respectively.

In the second logistic regression, the relationship between airflow obstruction and the risk of lung cancer was no longer significant after adjusting for visual emphysema. However, visual emphysema remained strongly associated with lung cancer risk after adjusting for COPD GOLD classification (OR, 5.4; 95% CI, 2.6–11.4; p<0.0001). Additionally, the risk of lung cancer was higher as the emphysema severity increased according to the NETT classification [OR 5.2 (95% CI: 2.4–10.9), 8.7 (95% CI: 2.7–27.9) and 12.5 (95% CI: 1.6–99.9), for categories 1, 2 and 3 respectively].

**Fig 1:**
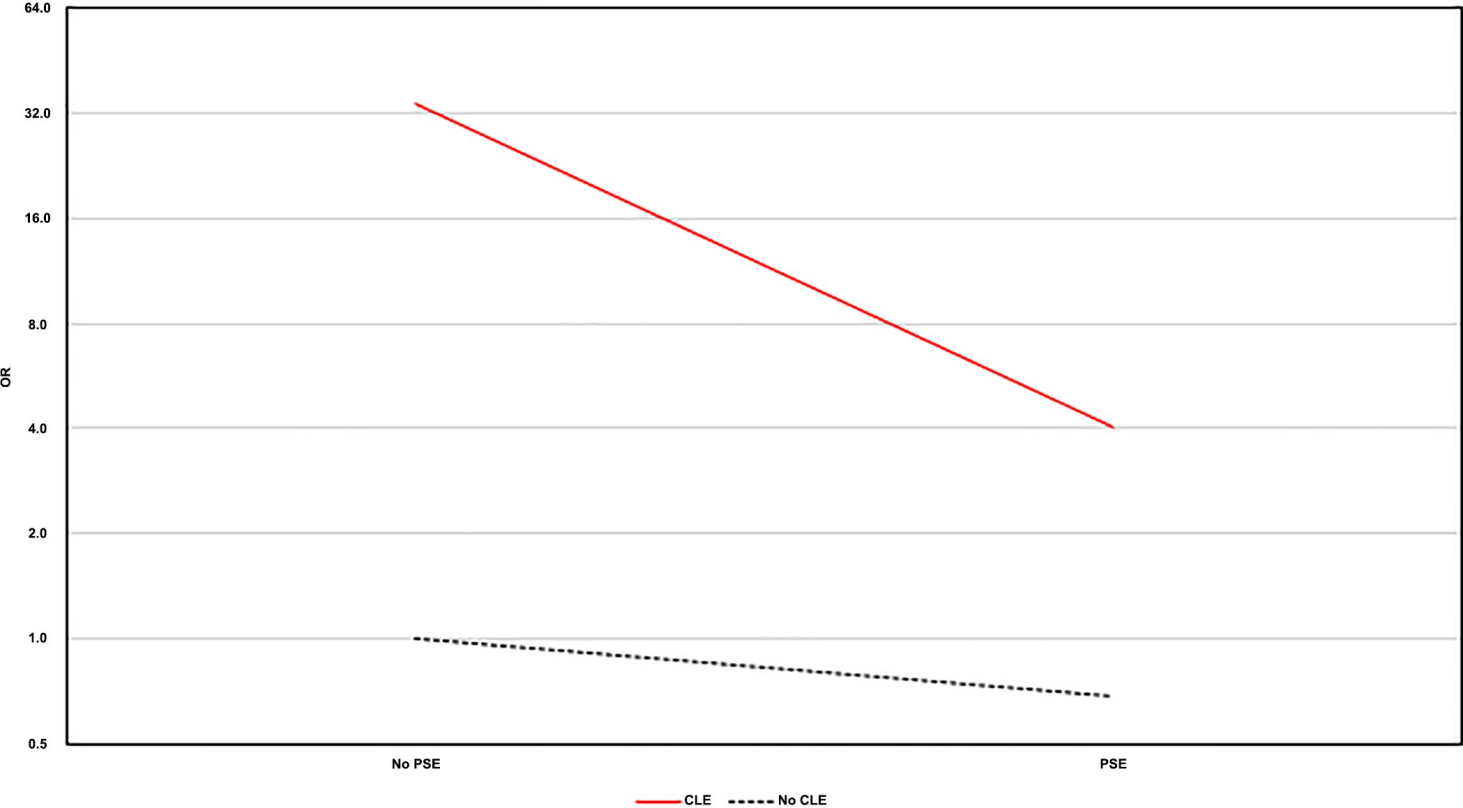
Odds ratios (OR) of the differents emphysema subtypes and lung cancer in the interaction model. This figure represents the ORs of the different emphysema subtypes with lung cancer. As it is shown, CLE (centrilobular) alone has the highest risk to develop lung cancer which dramatically decreases with the added presence of PSE (paraseptal). Surprisingly, PSE alone is not giving a differential risk of lung cancer.

To explore the relationship between emphysema subtypes and lung cancer risk, patients were grouped according to the emphysema subtype. Afterwards, an interaction model was completed to correctly assess this relationship. In comparison to patients without emphysema, the risk of lung cancer in patients with CLE alone was 34-fold greater (OR = 34.27, 95% CI: 25.48–99.31; p<0.000), whereas in patients with concomitant PSE the OR was 4.02 (95% CI: 3.61–34.97). Surprisingly, PSE alone did not show a clear risk of lung cancer with an OR of 0.68 (95% CI: 0.54–2.64; p<0.635). This can be seen better through the Fig 1.

### Associated data

Data is available on Figshare (https://doi.org/10.6084/m9.figshare.8046461.v1).

## Discussion

Our study contributes to existing evidence linking emphysema with lung cancer risk, and goes one step further by refining our knowledge of the risk associated with emphysema subtypes. [5,6,13,14] The finding that smokers with CLE are at greatest risk in comparison with those with PSE seems to point to a phenotypic predisposition to lung cancer related to emphysema distribution. We also confirmed previous reports suggesting that airflow obstruction is only associated with a higher risk for lung cancer on univariate analysis, but not after adjusting for the presence of emphysema.[3,4,24] This suggests that most of the lung cancer risk attributed to COPD may be due to the presence of emphysema.[5,6] Since many patients with emphysema do not have AO, it is important to emphasize this finding, just as current guidelines make therapeutic distinctions based on COPD phenotypes recommending different treatments for the emphysema and chronic bronchitic phenotypes.[25] A recent study by Carr *et al*., has also refined our understanding of the lung cancer risk associated with COPD by focusing on important differences in the features of patients with COPD that condition lung cancer risk.[26] In their study, COPD and its severity, respiratory exacerbations, and of course visual emphysema were independent predictors of lung cancer. As our understanding of the associations between COPD, emphysema and lung cancer grows, so does the conviction that risk is linked to specific phenotypes.

To the best of our knowledge, no previous studies have assessed the association between lung cancer and emphysema stratified by emphysema subtype. That CLE is associated with an increased risk for lung cancer makes biological sense since it is the subtype of emphysema more closely associated with smoking.[27,16] Moreover, CLE occurs more frequent in the upper lobes and in the right lung, just as cancer does.[16,28] The association between cigarette smoking and CLE has been attributed to systemic chronic inflammation and lung repair mechanisms unique to the exposure and perhaps a genetic predisposition. Patients with CLE have higher white blood cell counts [16] and a unique protease-antiprotease balance, characterized by a higher expression of matrix metalloproteinase 9 (MMP9) and transforming growth factor beta 1(TGFB1).[29] The former has been linked to both emphysema [30,31] and cancer [32,33]. In contrast to CLE, patients with PSE are often free of respiratory symptoms and are generally spared of the functional decline typically seen in patients with CLE.[16] PSE occurs in the absence of tobacco exposure and may depend on age and genetic susceptibility. Furthermore, PSE has been associated with the inhibitor of metalloproteinases 2 (TIMP2) and tumor necrosis factor.[29] The former is a tyrosine kinase receptor inhibitor of growth factor-mediated proliferation in both normal and tumor cells [34]. The activation of TIMP2 in tumor cells inhibits tumor cell growth *in vivo* and suppresses epithelial-to-mesenchymal transition (EMT), which is associated with tumor characteristics of aggressiveness and metastatic potential [35]. Therefore, it is likely that emphysema subtypes represent different disease phenotypes with implications beyond characteristic imaging findings, representing different genetic susceptibilities, molecular mechanisms, exposure to toxins, and clinical manifestations including risk of lung cancer.

Our study also found a linear trend between emphysema severity as measured by the NETT classification and lung cancer. Other studies have found an all-or-none effect.[5–7] For example, Wilson *et al*. found that lung cancer risk was greater for individuals with mild emphysema, followed by those with moderate or severe emphysema, and those with traces of emphysema.[6] Similarly, Li *et al* [7] found that lung cancer risk did not increase with emphysema severity (OR = 3.33; 95% CI: 2.30–4.82, and OR = 3.80; 95% CI: 2.78–5.19, for ≥10% and ≥5% emphysema, respectively). In a lung cancer screening cohort of 9,047 subjects from New York [36], the presence of emphysema, independently of its severity, predicted death from lung cancer (OR = 3.2; 95% CI: 1.5–6.7).

## Limitations

Our study is limited by group differences insofar as there were more active smokers in the group with lung cancer compared to controls. However, we made every attempt to correct for this potential bias by adjusting our analysis for pack-years smoked (matching criterion) and smoking status. Another limitation may be the predominance of male patients in our study (83% in cases and controls) as compared to other studies (*Wilson et al*.: 51.4%, *De Torres et al*.: 74%) [5,6]. This is important as women have been shown to demonstrate less radiographic evidence of emphysema than men.

## Conclusions

Our study shows that visually assessed CLE, and not PSE, is strongly associated with an increased risk of lung cancer. We believe our findings refine our current understanding of the risks associated with emphysema and contribute to a growing body of knowledge suggesting that emphysema subtypes represent specific phenotypes conditioned by genetic susceptibility, toxin exposure, molecular mechanisms and ultimately divergent clinical outcomes, which may have implications for lung cancer screening and follow up.
